# The effect of COVID-19 pandemic on perioperative factors: data from the Swedish Perioperative Register

**DOI:** 10.1186/s13741-023-00340-0

**Published:** 2023-09-16

**Authors:** Karuna Dahlberg, Sandra Månsson, Sara Lyckner, Lenita Lindgren, Fredrik Alm

**Affiliations:** 1 Faculty of Medicine and Health, School of Health Sciences, Örebro University, 701 82 Örebro, Sweden; 2 Department of Perioperative Medicine and Intensive Care, Karolinska University Hospital, Stockholm, Sweden; 3 Department of Anesthesia and Intensive Care, Mälarsjukhuset, Eskilstuna, Sweden; 4 Department of Nursing, Umeå University, Umeå, Sweden

**Keywords:** COVID-19 pandemic, Perioperative period, Postoperative outcomes, Anesthesia, Surgical interventions

## Abstract

**Background:**

The COVID-19 pandemic has affected healthcare organizations in many areas. The aim of this study was to describe surgical interventions, anesthesia, and postoperative outcomes in adult patients during the first wave and 1 year into the COVID-19 pandemic in Sweden, and to compare these outcomes with outcomes during the same period the year before the pandemic.

**Methods:**

Data were collected from the Swedish PeriOperative Register, and included 417, 233 perioperative registration of patients ≥ 18 years old between period 1 (March–June 2019), period 2 (March–June 2020), and period 3 (March–June 2021).

**Results:**

Compared with pre-pandemic (period 1), the number of surgical interventions decreased by 28% in the first wave (period 2); 1 year into the pandemic (period 3), the number of interventions was still 7.5% lower than pre-pandemic. The largest drops between periods 1 and 2 were noted in the specialties of ear, nose, and larynx surgery, – 55.6%; teeth, jaws, mouth, and pharynx surgery, – 45.0%; endocrine system surgery, – 38.8%. The number of acute surgeries remained stable during all three periods. Volatiles were more frequently used for the maintenance of general anesthesia in period 2 than in either period 1 or 3 (*p* < 0.001). Minor differences were noted throughout the periods in postoperative nausea and vomiting as well as postoperative pain.

**Conclusions:**

The COVID-19 pandemic has had an impact on perioperative care in Sweden. During the first wave of the pandemic, the number of surgical interventions decreased, but the number of acute surgeries remained stable compared with pre-pandemic numbers. Perioperative organizations have had and will continue to have challenges handling the increased number of patients needing perioperative care.

## Background

At the beginning of 2020, the COVID-19 pandemic resulted in an unexpected increase in the number of patients needing hospital and intensive care. As a result, in Sweden as in many other countries, handling the massive inflow of patients disrupted many routine care pathways. Perioperative care was especially affected, since resources were dedicated to intensive care (Søreide et al. 2020). In Sweden, most of the specialized perioperative teams, for example, comprising registered nurse anesthetists and anesthesiologists, were relocated to the intensive care units (ICUs), and even operating rooms and post-anesthesia care units (PACUs) were converted to ICUs. The greatest challenges were access to staff, equipment, and common drugs (Gerdin 2021). Priorities had to be made to create maximum capacity, and the National Board of Health and Welfare in Sweden published policy guidelines for medical priorities, including surgery (The National Board of Health and Welfare in Sweden 2020). As a result, the number of performed surgical procedures decreased drastically (Holmström et al. 2023) and regional healthcare policies were formulated to equalize the distribution of crucial drugs.

Local alterations in perioperative care were made to divert sedatives and analgesics for ICU patients (Gerdin 2021). Routines and guidelines for the remaining perioperative patients were frequently adapted to accommodate the prevailing circumstances (Søreide et al. 2020; Shuman et al. 2020), for example, by choosing other modes of administrating anesthesia (Ferrière et al. 2021).

The types of anesthetic agents and analgesics used affect several postoperative symptoms and complications, such as pain and postoperative nausea and vomiting (PONV), which can affect patients’ wellbeing and recovery after surgery (Tateosian et al. 2018). We hypothesized that there would be a significant change in the number of surgical interventions, anesthesia techniques, and postoperative symptoms due to staff rotation and alterations of perioperative routines during the first wave of the COVID-19 pandemic. The objective of this study was therefore to describe surgical interventions, anesthesia, and postoperative outcomes in adult patients during the first wave and 1 year into the COVID-19 pandemic in Sweden, and to compare these outcomes with outcomes during the same period the year before the pandemic.

## Methods

This is a retrospective cross-sectional study based on register data.

### Data collection

Data were collected from the Swedish PeriOperative Register (SPOR). SPOR is a national registry in Sweden established in 2011. It covers the complete perioperative process and can be used to increase perioperative quality. SPOR data can be used for comparison, evaluation, research, and the improvement of anesthesia and surgical quality (Chew et al. 2015). In 2019, 2020 and 2021, 78 settings (settings located at University Hospitals *n* = 12, county hospitals *n* = 24, smaller county hospitals *n* = 42) reported to SPOR, encompassing almost all patients in perioperative care in Sweden. Data are automatically transferred from local patient journals to the registry. SPOR has been validated against local databases and has demonstrated high accuracy (Holmström et al. 2023).

The inclusion criteria for this study were adult patients ≥ 18 years old who were registered in SPOR in the periods March–June 2019, March–June 2020, and March–June 2021. Variables included in this study were sex, age, hospital, American Society of Anesthesiologists (ASA)—class, type of surgical intervention, elective/acute surgery, length of surgery, type of anesthesia, postoperative pain, and PONV. Pain was measured as NRS or VAS 0–10 and PONV was measured as yes/no/vomiting.

### Data analysis

Outcomes from three time periods were analyzed. The pre-pandemic period (period 1) included data collected in March–June 2019. Data from the first wave of the COVID-19 pandemic (period 2) were collected in March–June 2020, and data from 1 year into the pandemic (period 3) were collected in March–June 2021. The first wave in this study was based on the massive increase of patients needing intensive care and the spread of the virus in the community in Sweden during March 2020. The number of patients needing intensive care increased in the following months and then decreased drastically in June 2020 (Gerdin 2021). Not all settings in Sweden report postoperative data to SPOR, so postoperative outcomes were only analyzed in settings where > 80% coverage of reporting postoperative pain (NRS) in patients recovering from general anesthesia (GA).

Surgical interventions were categorized according to the classification of the National Board of Health and Welfare in Sweden. The categorization is based on functional–anatomic body systems (The National Board of Health and Welfare in Sweden n.d.). All types of surgical interventions that could be categorized were included, also minor surgical procedures, therapeutic and investigative procedures. Unspecified procedures and unspecified region in the body were categorized as missing.

Descriptive data are presented as frequencies, percents, means, and standard deviations (SDs), as appropriate. Differences between the three periods were analyzed using the chi-squared test for categorical data, the Kruskal–Wallis test for ordinal data, and one-way ANOVA for continuous data. A *p* value < 0.01 was considered significant. IBM SPSS Statistics 27 was used for analyses.

## Ethical considerations

The study follows the principles outlined in the Declaration of Helsinki and its amendments. The study was approved by the Swedish Ethical Review Authority (Ref. number: 2021–04048). According to Swedish legislation, patients should be informed of research and quality registries and their use in research. All patients have the right to refuse participation in these registries. The data from SPOR were anonymized, and it was impossible to identify individual patients in the data.

## Results

**Fig. 1:**
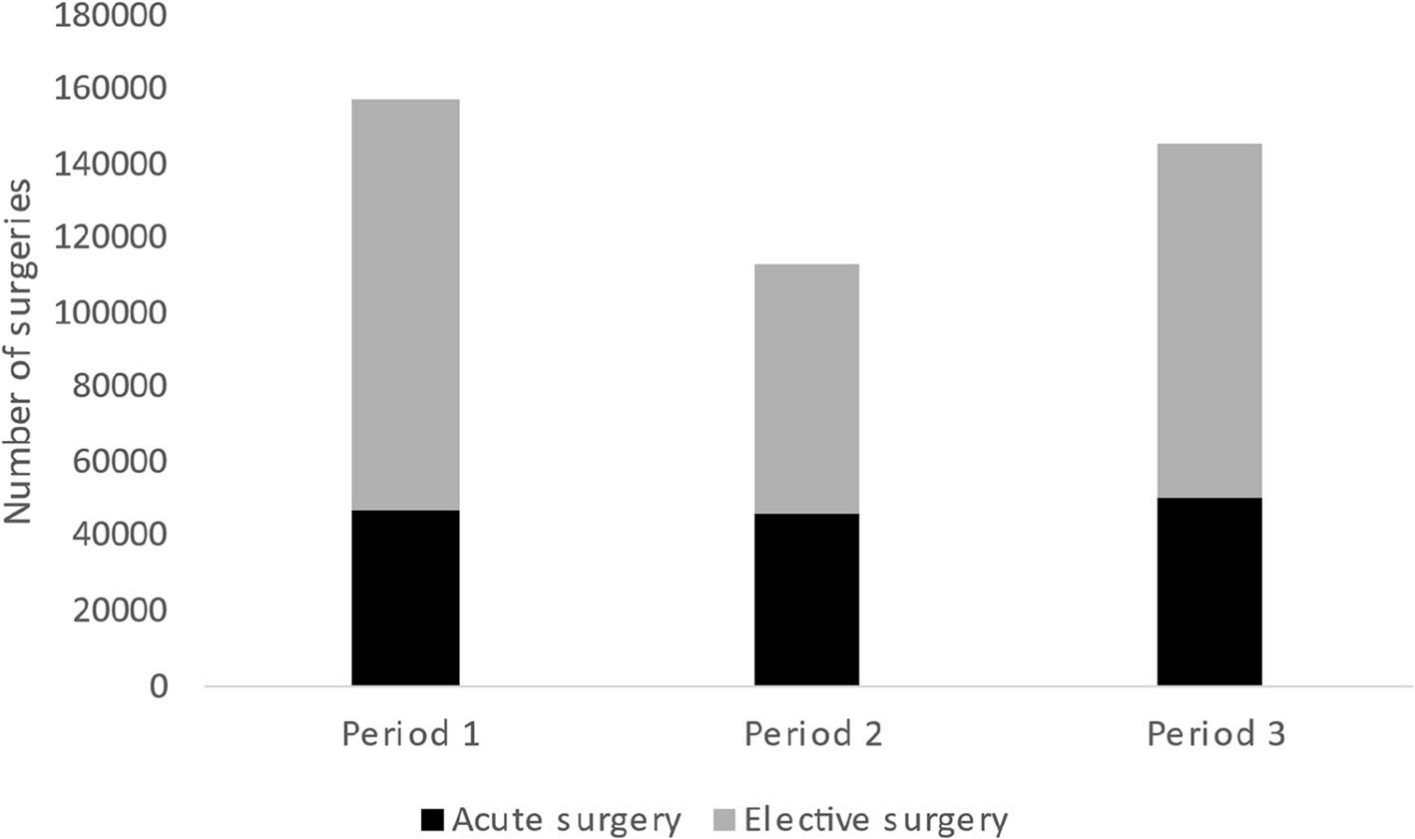
Proportion of acute and elective surgery during the three time periods. Period 1 = pre-pandemic period, March–June 2019; period 2 = first wave of the COVID-19 pandemic in Sweden, March–June 2020; period 3 = 1 year into the pandemic, March–June 2021

During the three time periods (pre-pandemic period = period 1: March–June 2019, First wave = period 2: March–June 2020, and 1 year into the pandemic = period 3: March–June 2021), 417,233 surgeries were carried out in 78 settings. From the pre-pandemic period 1 to the first wave of the COVID-19 pandemic (period 2), the total number of surgeries decreased by 28%. One year into the pandemic (period 3), the total number of surgeries was 7.5% lower than in the pre-pandemic period 1. There was a significantly higher proportion of acute surgeries performed during the first wave (period 2) (40.7% acute surgeries) than in either pre-pandemic period 1 (29.8% acute surgeries) or period 3 (34.9% acute surgeries). The number of acute surgeries remained stable during all three periods (Table 1, Fig. 1).

**Table 1:** Characteristics of surgical interventions during the three periods (*n* = 417,233)

	Missing	Period 1	Period 2	Period 3	*p* value
Number of surgeries		157,783	113,570	145,880	
Mean age, year	4 (< 0.00%)	58.1	57.7	58.7	< 0.001^a^
Sex, *n* (%)	1485 (0.36%)				0.12^b^
Female		88,989 (56.5)	63,003 (56.1)	82,041(56.3)	
Male		68,602(43.5)	49,362 (43.9)	63,751 (43.7)	
ASA 1	80,423 (19.3%)	33,856 (25.8)	22,152 (24.4)	27,527 (24.1)	< 0.001^c^
ASA 2		61,583 (46.9)	40,887 (44.9)	52,402 (45.8)	
ASA 3		32,131 (24.4)	24,364 (26.8)	30,287 (26.5)	
ASA 4		3715 (2.8)	3440 (3.8)	4044 (3.5)	
ASA 5		101 (0.1)	96 (0.1)	118 (0.1)	
ASA 6		40 (0.0)	29 (0.0)	38 (0.0)	
Acute surgery, *n* (%)	331 (<0.1%)	47,080 (29.8)	46,228 (40.7)	50,742 (34.9)	< 0.001^b^
Elective surgery, *n* (%)		110,702 (70.2)	67,333 (59.3)	94,817 (65.1)	
Surgery duration, mean, h:min:sec	0	1:11:23	1:14:00	1:12:08	< 0.001^a^

*Period 1* = pre-pandemic period, March–June 2019; *Period 2* = first wave of the COVID-19 pandemic in Sweden, March–June 2020; *Period 3* = 1 year into the pandemic, March–June 2021

^a^One-way ANOVA

^b^Chi-square

^c^Kruskal–Wallis test

### Surgical specialty

The most common surgical specialty during all three periods was surgery of the musculoskeletal system, followed by digestive system and spleen surgery. The surgical areas that experienced the largest proportional drop in number of surgical interventions between pre-pandemic period 1 and the first wave of the COVID-19 pandemic (period 2) were: ear, nose, and larynx surgery, – 55.6%; teeth, jaws, mouth, and pharynx surgery, – 45.0%; endocrine system surgery, – 38.8%; musculoskeletal system surgery, – 36.0%; and female genital organs surgery – 34.4% (Table 2).

**Table 2:** Surgical specialties and number of surgical interventions performed during period 1 (pre-pandemic period, March–June 2019), period 2 (first wave of the COVID-19 pandemic in Sweden, March–June 2020), and period 3 (1 year into the pandemic, March–June 2021)

Surgical specialty	Period 1 *n*	Period 2 *n*	Period 3 *n*	Difference in percent between periods 1 and 2	Difference in percent between periods 1 and 3
D	2776	1233	1947	– 55.6	– 29.9
E	3515	1932	3063	– 45.0	– 12.9
B	1450	888	1324	– 38.8	– 8.7
N	43,227	27,682	36,096	– 36.0	– 16.5
L	11,540	7575	9685	– 34.4	– 16.1
A	8389	5666	7227	– 32.5	– 13.9
J	28,919	20,681	26,977	– 28.5	– 6.7
C	9655	7056	11,804	– 26.9	+ 22.3
K	11,382	8614	11,125	– 24.3	– 2.3
H	4504	3485	4458	– 22.6	– 1.0
F	5453	4482	4956	– 17.8	– 9.1
P^a^	6587	5505	2558	– 16.4	NA
Q	7259	6089	7041	– 16.1	– 3.0
M	8914	9042	9506	+ 1.4	+ 6.6
G	1257	1518	1503	+ 20.1	+ 19.6
V^a^	0	0	4014	NA	NA

Surgical specialties are ordered in descending order based on the largest drop in the number of surgical interventions between pre-pandemic period 1 and the first wave of the COVID-19 pandemic (period 2)

Categorized according to the classification of the National Board of Health and Welfare in Sweden based on functional–anatomic body systems: *A* = nervous system, *B* = endocrine system, *C* = eye and adjacent structures, *D* = ear, nose, and larynx, *E* = teeth, jaws, mouth, and pharynx, *F* = heart and major thoracic vessels, *G* = chest wall, pleura, mediastinum, diaphragm, trachea, bronchus, and lung, *H* = mammary gland, *J* = digestive system and spleen, *K* = Urinary system, male genital organs, and retroperitoneal space, *L* = female genital organs, *M* = obstetric procedures, *N* = musculoskeletal system, *P* = peripheral vessels and lymphatic system (V^a^ from 2021), *Q* = skin

Unspecified procedures and unspecified region in the body categorized as missing *n* = 4424. Anesthetic procedures such as inserting central venous catheter or intravenous cannula are not included *n* = 3250

### Anesthesia

For 350,925 patients (84.1%), the type of anesthesia was reported to SPOR. Of these patients, 240,163 underwent GA (68.4%). During the first wave of the COVID 19 pandemic (period 2), significantly fewer (32.6% decrease) patients underwent GA than in the pre-pandemic period 1, and this remained the pattern 1 year into the COVID-19 pandemic (period 3) (11.3% decrease). In a significantly higher proportion of surgeries, volatiles were used to maintain GA during period 2 than in either period 1 or 3 (Table 3). The same significant differences were also seen when acute and elective surgeries were analyzed separately.

**Table 3:** General vs. regional anesthesia and maintenance of general anesthesia with volatiles vs. intravenous anesthesia

	Missing, *n* (%)	Period 1, *n* (%)	Period 2, *n* (%)	Period 3, *n* (%)	*p* value
General anesthesia	66,308 (15.9)	93,798 (69.5)	63,208 (67.3)	83,157 (68.1)	< 0.001^a^
Regional anesthesia		36,598 (27.1)	28,079 (29.9)	38,153 (31.3)	
Other		4591 (3.4)	2564 (2.3)	777 (0.6)	
Maintenance of GA					< 0.001^a^
Volatiles	7199 (3.0)	38,457 (42.5)	37,059 (61.1)	37,918 (46.3)	
Intravenous		51,940 (57.5)	23,597 (38.9)	43,993 (53.7)	

*Period 1* = pre-pandemic period, March–June 2019; *Period 2* = first wave of the COVID-19 pandemic in Sweden, March–June 2020; *period 3* = 1 year into the pandemic, March–June 2021; *GA* general anesthesia

^a^Chi-square

### Postoperative outcomes

Postoperative outcomes were only analyzed in settings with > 80% coverage of postoperative pain (NRS) in patients recovering from GA. Thirty-three of 78 settings met this criterion, giving a total of 79,616 cases (33.2% of the patients that underwent GA).

There were significant differences in the postoperative outcomes PONV and pain when comparing the three periods, although the differences might not be clinically relevant. A slightly higher proportion of patients experienced PONV during the first wave of the COVID-19 pandemic (period 2). Regarding pain, a slight improvement in pain was seen during the first wave of the COVID-19 pandemic (period 2) (Table 4).

**Table 4:** Overview of postoperative outcomes in the PACU (*n* = 79,616)

	Missing, *n* (%)	Period 1	Period 2	Period 3	*p* value
PONV, *n* (%)	6306 (7.9)	2790 (9.4)	1918 (10.7)	2485 (9.7)	< 0.001^a^
No PONV, *n* (%)		26,900 (90.6)	16,006 (89.3)	23,211 (90.3)	
Pain NRS, highest, mean (SD)	7452 (9.4)	2.46 (2.97)	2.32 (2.89)	2.38 (2.92)	< 0.001^b^
Pain NRS, after 1 h, mean (SD)	13,692 (17.2)	1.87 (2.83)	1.79 (2.74)	1.89 (2.78)	0.003^b^
Pain NRS, at discharge, mean (SD)	10,588 (13.3)	1.07 (1.47)	1.04 (1.48)	1.06 (1.47)	0.107^b^

*Period 1* = pre-pandemic period, March–June 2019; *Period 2* = first wave of the COVID-19 pandemic in Sweden, March–June 2020; period 3 = 1 year into the pandemic, March–June 2021. *NRS* = numeric rating scale

^a^Chi-square

^b^One-way ANOVA

## Discussion

This study finds a reduction in the number of surgical interventions, the number of elective surgeries, and the intravenous maintenance of anesthesia during the first wave of the COVID-19 pandemic. The largest decreases in the proportion of surgeries were seen in ear, nose, and throat (ENT) and oral surgery. The number of performed surgical interventions had not recovered to pre-pandemic levels 1 year into the pandemic.

The pandemic had an impact on perioperative care in many countries, and during 2020 it was predicted that about 80% of surgeries would be postponed during the peak of the COVID-19 pandemic (COVIDSurg Collaborative 2020). A decrease in the number of surgeries was seen during the COVID-19 pandemic in the Europe (Shaw et al. 2022), the USA (Zhong et al. 2021), Japan (Okuno et al. 2021), and North and South America (Beninato et al. 2022), and a decrease in the number of elective surgeries was also described (Beninato et al. 2022). The ratio between the numbers of elective and acute surgeries in south-east Queensland, Australia was similar to that found here: the proportion of elective surgeries decreased from 65.18% in March–April 2019 to 58.96% during the first wave of the COVID-19 pandemic (March–April 2020) (Fowler et al. 2021), compared with our finding of a slightly greater decrease from 70.2 to 59.3%. Also, in south-east Queensland, the surgical specialties experiencing the largest decrease were similar to those found here: a decrease from 7.2 to 4.6% was seen for maxillo-facial/dental/ENT surgery and from 6.1 to 3.6% for ophthalmologic surgery (Fowler et al. 2021). Similar decreases in ophthalmologic and ENT surgeries were also described in Japan during the first wave of the COVID-19 pandemic (Okuno et al. 2021). In our results, we also identified an increase in interventions involving chest wall, pleura, mediastinum, diaphragm, trachea, bronchus, and lung during the first wave of the pandemic. It is possible that this increase could be related to the rise in patients developing acute respiratory distress syndrome needing intensive care and mechanical ventilation (Meyer et al. 2021).

We could identify a shift in the choice of maintenance anesthesia to volatiles during the first wave of the COVID-19 pandemic. This can be explained by the shortage of drugs caused by the increased number of patients needing sedation in ICUs. To our knowledge, this shift has not been described before. Shaw et al. described a shift in anesthetic techniques from GA to regional/local anesthesia in patients undergoing hand surgery, and noted that more surgeries were performed in minor OR settings (Shaw et al. 2022). Takazawa et al. identified a decrease in GA during the first wave of the COVID-19 pandemic (Takazawa et al. 2021). We also found a reduction in GA in our study, but mainly due to the decrease in the number of surgeries.

The number of acute surgeries remained stable during the three studied periods, indicating that the perioperative organizations were able to maintain acute perioperative care. This has also been indicated earlier by Holmström et al. (Holmström et al. 2023). We could not identify any large differences in postoperative outcomes between the pre-pandemic period and the first wave of the pandemic. Even though a higher proportion of volatiles was in use, the incidence of PONV increased only slightly. Possibly, the above results indicate that anesthesiology settings and PACUs are resilient organizations that could maintain acceptable postoperative care despite the vast workloads and changes to routines imposed on these units during the COVID-19 pandemic. Organizations that have the capacity to maintain an acceptable level of functioning have a higher organizational resiliency (Barasa et al. 2018). One year into the pandemic (period 3), the number of surgeries had recovered somewhat but had still not reached the pre-pandemic level. This means that a large number of patients have waited and are still waiting for surgeries in Sweden.

The present results highlight the impact that the pandemic has had on perioperative care and on different surgical specialties. It also highlights the need for sufficient resources to restore the perioperative organizations so they can handle the remaining care backlog. The staff who coped with the large inflow of critically ill patients and the new constraints during the COVID-19 pandemic are the same staff who must now work to provide safe and high-quality perioperative care to a large number of patients.

### Limitations

This study has several limitations. The large volume of data analyzed means that even small differences can be statistically significant. Therefore, the results need to be interpreted from the perspective of what is clinically relevant.

SPOR does not cover all surgical interventions in Sweden. Not all settings report postoperative data and outcomes to SPOR, and we found quite a large proportion of missing data. Only 33 settings were included in the analysis regarding postoperative outcomes. It is likely that other results could have been identified if all settings reported postoperative outcomes and were included in the analysis. There could also be some errors in the reporting of postoperative outcomes. All postoperative outcomes are manually reported by the staff working in the PACUs. It is possible that, due to the increased workload and staff changes during the pandemic, such reporting was deprioritized and that false values were reported. We therefore recommend that our results should be interpreted with that in mind.

Other postoperative outcomes registered in SPOR, such as postoperative complications, are of interest. These variables are still not reported by all settings to SPOR, and as we found a large volume of missing data for these variables, we did not include them in this study.

In present study, we report register data from SPOR that only includes perioperative data. Future studies should investigate whether there was an increase in unexpected admissions and readmissions related to the decreased number of performed surgeries and the increased proportion of volatiles used.

## Conclusion

The COVID-19 pandemic has had an impact on perioperative care in Sweden. During the first wave of the pandemic, there was a decrease in the total number of surgical interventions, although the number of acute surgeries remained stable compared to pre-pandemic numbers. There were differences in the degrees to which various surgical specialties were affected. An increase was seen in the proportion of volatiles used during GA. The perioperative organizations had and will continue to have challenges handling the increased number of patients needing perioperative care.

## Data Availability

The datasets generated and analyzed is this study are not publicly available due to Swedish legislation, but are available from the corresponding author on reasonable request.
